# The current status of suicide and self-injury in eating disorders: a narrative review

**DOI:** 10.1186/s40337-014-0019-x

**Published:** 2014-07-11

**Authors:** Katrina Kostro, Jessica B Lerman, Evelyn Attia

**Affiliations:** Columbia University Medical Center, New York, NY USA; Weill Cornell Medical College, New York, NY USA

**Keywords:** Anorexia nervosa, Bulimia nervosa, Binge eating disorder, Eating disorders, Suicidality, Suicide, Mortality, Self-injury, Non-suicidal self-injury, Review

## Abstract

The aim of this paper is to review recent literature on suicide and self-injury in eating disorders (ED) including anorexia nervosa (AN), bulimia nervosa (BN), and binge eating disorder (BED). Among psychiatric diagnoses, EDs are associated with increased mortality rates, even when specialized treatment is available. Of the mortalities that are reported in individuals with EDs, suicide is among the most commonly reported causes of death. Additionally, suicidal and non-suicidal self-injurious behaviors occur frequently in this clinical population. A literature search was undertaken using the databases of Medline/PubMed and PsycInfo to identify papers describing suicidality in individuals with ED diagnoses. The authors identified studies and review articles published between 2005-2013 (inclusive) that describe the relationship between EDs and suicide, and associated behaviors including self-injurious behaviors, or non-suicidal self-injury (NSSI). The initial search resulted in 1095 papers that met the *a priori* search criteria. After careful review, 66 papers were included. The majority of papers described clinical cohorts that were studied longitudinally. The diagnosis described most frequently in selected studies was AN. There are limited current data about the prevalence of suicide and NSSI among individuals with EDs. Among the published studies that focus specifically on the relationship between EDs and suicidality, most describe AN in more detail than other EDs. Nonetheless, rates of mortality, and specifically rates of suicide, are undeniably high in ED populations, as are the rates of self-harm. Therefore, it is critical for clinicians and caretakers to carefully evaluate these patients for suicide risk and to refer promptly for appropriate treatment.

## Background

Eating disorders (ED), including anorexia nervosa (AN) and bulimia nervosa (BN), are serious psychiatric illnesses with high rates of morbidity and mortality. Studies of clinical samples, mostly from specialized programs in Europe and North America, have described high rates of suicidal behaviors and completed suicides associated with EDs, and have helped increase clinical awareness about the seriousness of these psychiatric disorders [1–6]. A comprehensive review published in 2006 by Franko and Keel describes findings from 1985-2004 [7]. This review highlighted findings of high suicide rates among individuals with AN, and contrasted these with individuals with BN, whose rates of suicide did not appear to be elevated. Conversely, the review reported that non-lethal suicide attempts occurred in 25-35% of BN patients, compared with a rate of 3-20% of AN patients [7]. The review also described common clinical correlates of suicidality in EDs, including comorbid diagnoses such as depression, self-injurious behaviors such as bingeing and purging, a history of substance abuse, and a history of childhood abuse.

Considering the consistent finding of high risk of suicidality in this population, it is important for researchers and clinicians to continue to examine the risk factors and associations with the prevalence of suicidality in individuals with EDs. This narrative review aims to update the previous review by Franko and Keel [7], and to extend the scope to include non-suicidal self-injury (NSSI). The authors included NSSI in this review as these behaviors are commonly reported in EDs and were not included in many earlier reports of suicidality in this clinical population. The authors aimed to include publications that described all ED diagnosis groups, including binge eating disorder (BED) and eating disorder not otherwise specified (EDNOS), as many earlier reviews focused primarily on AN and BN. The authors aimed to describe the available data, including their quality and scope and to identify areas in need of additional study.

## Review

### Methods

The authors performed a literature search between July and December 2013, using the databases of Medline/PubMed and PsycInfo, to review papers published between January 1, 2005 and December 31, 2013, describing suicidality and self-injury in individuals with ED diagnoses. The start date for the search interval was selected because the most recently published comprehensive review on similar subject matter described studies published between 1985-2004. Twenty advanced searches were undertaken, combining ED key words with suicidality key words. The first and second key words of each search were required to appear in the article title or abstract. The ED key words used were: “eating disorders”; “anorexia nervosa”; “bulimia nervosa”; “binge eating disorder”; or “eating disorder not otherwise specified”. Each of the five ED terms was paired with a second key word of the following key words: “suicide”; “mortality”; “self-injurious behavior”; or “non-suicidal self-injury”. Additionally, the authors conducted four searches, combining the ED key words “avoidant/restrictive food intake disorder” and “ARFID” with each of the four selected second key words to identify any recent literature that may have been published in anticipation or soon following the release of DSM-5 in May 2013.

Articles were included in this review if studies included information about the relationship between suicidality or self-injury and individuals with ED diagnoses including AN, BN, BED, EDNOS and ARFID. Articles were excluded if they did not present data about suicidality or self-injury in EDs, or if they did not identify and discuss a specific relationship between EDs and suicidality or self-injury. Only articles in English were reviewed. The articles were reviewed by all authors of this paper.

### Results

#### Quality appraisal of included papers

**Figure 1:**
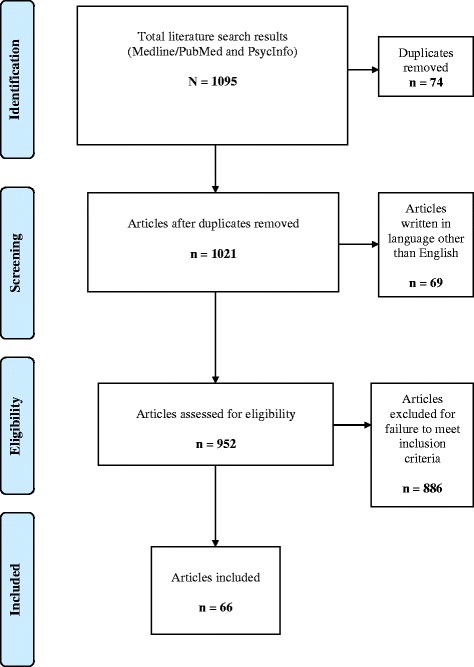
**Flow chart of included studies.**

The literature search of suicidality and EDs produced overlapping lists of published studies and review articles. The initial search resulted in 1095 papers that met the *a priori* search criteria (Figure 1). Seventy-four duplicate articles were removed, as were 69 articles written in languages other than English, and 886 papers were excluded due to failure to include data regarding suicidality in EDs. The authors ultimately identified 66 papers that met the inclusion criteria and present them in Table 1 including information about study type, sample size, age group and gender. The literature search results are highlighted in narrative text organized by ED diagnosis and then by general features relevant to risk of suicidality and EDs.

**Table 1:** **Papers describing suicide and/or self-injury associated with eating disorders**

**Author** ***N = 66***	**Study design** ***n = 70***	**Sample size** ***n = 54***	**ED diagnoses** ***n = 67***	**Age group** ***n = 55***	**Gender** ***n = 52***
Ahrén-Moonga, [8]	Clinical cohort with controls	105*	AN, BN	Adults	Females
Ajetunmobi, [1]	Community cohort	111504*	ED	Adults	Mixed
Anestis, [9]	Clinical cohort	127	BN	Adults	Females
Arcelus, [10]	Review/meta-analysis	-	AN, BN, EDNOS	-	-
Berkman, [11]	Review/meta-analysis	-	AN, BN, BED	-	-
Birmingham, [12]	Clinical cohort	954	AN, BN, EDNOS	Adults	Females
Bodell, [13]	Community cohort	364	AN, BN, EDNOS	Adults	Females
Bulik, [14]	Clinical cohort	432	AN, BN	Adolescents and adults	Mixed
Button [15]	Clinical cohort	1892	AN, BN, EDNOS	Adults	Females
Carano, [16]	Clinical cohort	80	BED	Adults	Mixed
Chen, [17]	Clinical cohort	135	AN, BN, BED	Adults	Females
Claes, [18]	Clinical cohort	70*	AN, BN	Adults	Females
Claes, [19]	Clinical cohort	130	AN, BN, EDNOS	Adults	Males
Crow, [20]	Clinical cohort	1885	AN, BN, EDNOS	Adults	-
Dancyger, [21]	Review/meta-analysis	-	AN, BN, EDNOS	Adolescents	-
Erdur, [22]	Clinical cohort	169*	AN	Adolescents and adults	Females
Favaro, [23]	Clinical cohort	934	AN, BN, ED	Adults	Females
Favaro, [24]	Clinical cohort	95	BN	Adults	-
Fedorowicz, [25]	Clinical cohort	968*	AN, BN, EDNOS	Adolescents and adults	Females
Fennig, [26]	Clinical cohort	46	AN, BN	Adolescents	Females
Fichter, [27]	Clinical cohort	103	AN	Adolescents and adults	Females
Fischer, [28]	Clinical cohort	80	BN	Adolescents	Mixed
Forcano, [29]	Clinical cohort with controls	172	AN	Adults	Females
Forcano, [30]	Clinical cohort	566	BN, EDNOS	Adults	Females
Foulon, [2]	Clinical cohort	304	AN	Adolescents and adults	Mixed
Franko, [5]	Clinical cohort	246	AN, BN	Adolescents and adults	Females
Franko, [7]	Review/meta-analysis	-	AN, BN	-	-
Fujimori, [31]	Clinical cohort with controls	200	ED	Adults	Females
Grucza, [32]	Community cohort	910	BED	Adults	Mixed
Gueguen, [33]	Clinical cohort	1009*	AN	Adults	Mixed
Guillaume, [34]	Clinical cohort with controls	1563*	AN, BN	Adults	Mixed
Harris, [35]	Review/meta-analysis	-	AN, BN, BED, EDNOS	-	-
Herpertz-Dahlmann, [36]	Review/meta-analysis	-	AN, BN, BED, EDNOS	-	-
Hoek, [37]	Review/meta-analysis	-	AN, BN	-	-
Huas, [38]	Clinical cohort	601*	AN	Adults	Females
Huas, [39]	Clinical cohort with controls	258*	BN	Adolescents and adults	Females
Latzer, [40]	Review/meta-analysis	-	AN	-	-
Liang, [41]	Clinical cohort with controls	316	AN, BN, EDNOS	Adolescents and adults	Females
Millar, [42]	Clinical cohort	524	AN	Adolescents and adults	Females
Muehlenkamp, [43]	Clinical cohort	422*	AN, BN, BED, EDNOS	Adults	Females
Muehlenkamp, [44]	Clinical cohort	131	BN	Adults	Females
Nickel, [3]	Community cohort	813	BN	Adolescents and adults	Females
Papadopoulos, [45]	Clinical cohort	6009*	AN	Adolescents and adults	Females
Papadopoulos, [46]	Clinical cohort	5251	AN	Adolescents and adults	Females
Peebles, [47]	Clinical cohort	1432	ED	Adolescents and adults	Mixed
Peterson, [48]	Clinical cohort	489	BN	Adolescents and adults	Females
Pisetsky, [4]	Clinical cohort	13035	AN, BN, BED	Adults	Females
Pompili, [49]	Review/meta-analysis	-	AN, BN	-	-
Preti, [50]	Review/meta-analysis	-	AN, BN, BED	-	-
Rigaud, [51]	Clinical cohort	484*	AN	Adolescents and adults	Mixed
Rosling, [52]	Clinical cohort	201*	AN, BN	Adolescents and adults	Mixed
Ross, [6]	Community cohort with controls	440	ED	Adolescents	Mixed
Ruuska, [53]	Clinical cohort	57	AN, BN	Adolescents	Females
Schroeder, [54]	Clinical cohort with controls	69	AN, BN	Adults	Females
Selby, [55]	Clinical cohort	787	AN	Adolescents and adults	Mixed
	Clinical cohort	249	AN	Adolescents and adults	Mixed
Signorini, [56]	Clinical cohort	2240	AN	Adults	Females
Silber, [57]	Review/meta-analysis	-	AN, BN	-	-
Smink, [58]	Review/meta-analysis	-	AN, BN, BED, EDNOS	-	-
Smink, [59]	Review/meta-analysis	-	AN, BN, BED, EDNOS	-	-
Smith, [60]	Clinical cohort	204	BN	Adolescents and adults	Females
	Community cohort	171	-	Adolescents and adults	Mixed
	Community cohort	467	-	Adolescents and adults	Mixed
	Community cohort	512	-	Adolescents and adults	Mixed
Steinhausen, [61]	Review/meta-analysis	-	AN, BN	-	-
Steinhausen, [62]	Review/meta-analysis	-	BN	-	-
Suokas, [63]	Clinical cohort with controls	2450	AN, BN, BED	adults	Mixed
Svirko, [64]	Review/meta-analysis	-	AN, BN, EDNOS	-	-
Tozzi, [65]	Clinical cohort	1021	AN, BN, EDNOS	Adolescents and adults	Mixed
Vansteelandt, [66]	Clinical cohort	59	AN, BN	Adults	Females

*Note*. All ED diagnoses are based on DSM-IV. The following abbreviations are used: AN = anorexia nervosa; BN = bulimia nervosa;

BED = binge eating disorder; ED = eating disorders, general; EDNOS = eating disorder not otherwise specified.

*inpatient.

The literature included variation regarding methodology, sample size, length of follow-up, and diagnostic criteria that may account for the variability among studies. Overall, the authors found that data on the prevalence of suicidality in EDs have focused mainly on AN. Interpretation of the more limited data about BN is difficult because information regarding initial ED diagnosis is rarely available and diagnostic cross-over from AN to BN is high, with estimated cross-over rates ranging from 20-50% [10]. Additionally, many studies examined predictors of poor outcome in EDs but did not specify predictors of mortality. Furthermore, the cause of patient deaths, including death by suicide, was not always readily available, and thus limited the ability to interpret study findings. Also, there were few studies assessing the cause of mortality in patients with BN, BED, and other EDs not otherwise specified.

#### Anorexia nervosa

Until recently, the majority of research regarding increased mortality risk conferred by EDs, including risk of suicidality, has focused on AN. Across ED diagnoses, recent studies examining the characteristics of suicide attempts among individuals with AN, BN, and individuals without EDs, found higher Standardized Mortality Ratios (SMR) in AN than in BN, ranging from 6.2 to 10.6 [34,39,52]. SMR represents the observed number of deaths divided by the expected number of deaths in a certain group, and thus represents the estimated risk of death in a group compared to individuals from the general population, matched by age and gender. A clinical cohort study published by Bulik et al. in 2008 reported suicide attempts approaching approximately 17% in the AN population [14]. According to Guillaume et al., the odds ratio (OR) for suicide attempts in AN compared to other ED diagnoses showed that AN patients were more likely to have made a serious attempt (OR 3.4, 95% CI 1.4-7.9) [34]. The results also showed that the AN group with suicide attempts had a higher expectation of dying (OR 3.7, 95% CI 1.1-13.5) based on scores from The Suicidal Intent Scale, a 15-item scale that assesses the severity of suicidal intention associated with suicide attempts [34]. Huas et al. concluded that in AN patients with a lifetime history of suicide attempt, risk of death was multiplied by 2.6 [38].

Reported rates of suicide attempts in AN are high, and there are also data confirming completed suicides in individuals with AN. Huas et al. reported that suicide was the second highest cause of death (17%, n = 7) in a clinical cohort with AN, 28.4% of which had committed at least one suicide attempt [38]. Papadopoulos et al. found that the SMR of observed deaths caused by suicide in a study population defined by having been admitted at least once for treatment of AN, compared to expected suicide deaths in the general population, was 13.6 (95% CI 10.9-16.8) [45]. The SMR was only exceeded in this sample by deaths due to psychoactive substance use (SMR 18.9, 95% CI 10.0-32.3) and those caused primarily by AN (SMR 650.0, 95% CI 462.2-888.6) [45]. In a study that analyzed eight years of follow-up data from 147 outpatients with AN, and combined these data with those from 10 additional studies that included follow-up data from other samples, Signorini et al. reported 5.3% overall mortality rate, including 1.2% death due to suicide [56]. In a meta-analysis of 36 studies published between 1966-2010 that included mortality rates in individuals with EDs, Arcelus et al. found a SMR of 5.9 for the AN sample, in which one in five individuals who died had committed suicide [10].

While overall mortality risk, including risk of death by suicide, appears to be significantly higher in AN, compared to risk in individuals with BN and BED [11], there are significant differences in rates of suicidality between AN subtypes: 7.4% of AN-restricting subjects reported at least one suicide attempt, whereas approximate rates are between 20 and 30% for other AN subtypes [36]. Selby et al. conducted a two-part cohort study investigating suicidality and associated “provocative behaviors” among AN subtypes including: restricting, purging, binge-purging, and history of BN [55]. The study found in both cohorts (n = 787 and replicated n = 249) that suicidal behavior in AN may be the result of these associated behaviors defined by the AN subtypes. Repetitive experience with behaviors such as binge eating and self-induced vomiting may be a route to suicidal behavior in bingeing and/or purging subtypes, while the exposure to pain through starvation from restriction may lead to suicidal behavior in restricting subtypes [29,51,55].

Other factors that have been specifically examined in relation to suicidality in populations with AN include patients’ age at assessment, pregnancy and gender. Arcelus et al. found that the age of the patient at the time of ED assessment is a significant predictor of mortality, including death by suicide [10]. Pregnancy has also been examined in relation to suicidality and AN. In a population-based analysis, 5251 females with AN were evaluated, comparing suicide rates between a parous and a nulliparous group [46]. Suicide was the most common cause of death in both groups. The SMR for suicide in the parous group was 6.3 (95% CI 2.7-12.3) compared to 16.1 in the nulliparous group (95% CI 1.8-21.4), and in fact, childbearing was associated with 65% lower general mortality in AN [46]. While few studies evaluate AN in the male population due to the small sample sizes, Gueguen et al. conducted a longitudinal cohort study comparing 23 men with AN to 601 women with AN and found that men with AN were less likely to have a history of a suicide attempts compared to women with AN (4% vs. 29%; p = 0.01) [33].

#### Bulimia nervosa

Previously, data have shown that mortality rates in general are lower in populations with a diagnosis of BN than in populations with a diagnosis of AN, but few data have specified rates of suicidality in BN. For example, the meta-analysis from Arcelus et al. and a longitudinal study from Franko et al. both examined the mortality rates for BN: Arcelus et al. found a weighted mortality rate of 1.7 (95% CI 1.09-2.44) per 1000 person-years, compared to a weighted mortality rate of 5.1 for AN [10], and Franko’s findings were similar [5], but neither of these reports mentioned suicide as a cause of death in the BN population. One study that did look at suicide in BN found that characteristics of suicide attempts did not significantly differ between BN and controls with no ED diagnosis [34].

With regard to completed suicides in BN, findings are mixed. Rates of suicide in both the BN and control samples from Guillaume et al. were altogether lower than in patients with AN [34]. A meta-analysis by Preti et al. reported that in BN, suicide as the cause of death was present in roughly the same proportion as that seen in AN [50]. Crow and colleagues reported a larger percentage of death due to suicide in a BN sample (0.9%) versus an AN sample (0.6%) [20]. Overall, however, the literature indicates that the number of completed suicides among BN appears to be low in comparison to AN.

Some studies have focused on suicidal ideation and suicide attempts, as opposed to completed suicides, and have found that, consistent with the earlier findings of Franko and Keel, suicide attempts are more prevalent in BN populations than in AN populations [7]. Investigating the risk for suicidality between patients with AN, BN and EDNOS, Bodell and colleagues found that BN was significantly associated with suicidality independent of risk predicted by comorbid disorders, while neither AN nor EDNOS were found to be uniquely associated with suicidality [13]. In a meta-analysis by Fedorowicz and colleagues, BN was most strongly associated with suicide attempts or suicidal ideation, followed by AN-binge/purge subtype [25]. Ahrén-Moonga and colleagues investigated the prevalence of suicide plans and/or thoughts, as well as history of suicide attempts in inpatients with EDs, and found a higher prevalence of suicide attempts associated with BN compared with AN (p < 0.02) [8]. Forcano et al. found a lifetime prevalence of suicide attempts to be 26.9% (n = 152, 95% CI 23.2-30.5) in a sample of 566 BN outpatients [30]. More recently published data from Smith et al. found that approximately one-third of women with a diagnosis of BN have had at least one suicide attempt [60]. There are fewer published data on adolescents with BN than on adults, but a diagnosis of BN in adolescents does appear to raise the risk of increased suicidality and self-harm. For example, in a study that looked at 80 adolescents with BN, 25% reported having attempted suicide or engaging in self-injurious behavior [28]. A study comparing adolescents with AN to adolescents with BN found that the BN group had significantly more suicidal ideation than the AN group [53].

#### Binge eating disorder and other categories of eating disorders

Less is known about the prevalence of suicidality in populations with a specific diagnosis of BED, or in individuals previously diagnosed with EDNOS according to DSM-IV, or other categories of illness described in the DSM-5 such as ARFID. BED was described in DSM-IV but identified as a formal diagnostic category only recently with the publication of DSM-5. The authors identified few studies that specifically examine suicide associated with BED. Among collected data from community samples, subjects with BED have shown elevated odds for suicide attempts [4,32]. In a thorough examination of all deaths reported among a clinical cohort of 2450 individuals with EDs and a control population, followed for up to 15 years (average length of follow-up was 8.7 years), Suokas et al. identified that four deaths occurred among the 171 individuals with broadly defined BED, one of which was due to suicide [63]. Pisetsky et al. reported that 19% of a sample that met criteria for a narrow diagnosis of BED, and 59% of a sample that met for a broad diagnosis of BED, had a lifetime prevalence of at least one suicide attempt [4]. No results were produced from literature searches using ED key words “avoidant/restrictive food intake disorder” or the acronym “ARFID”.

#### Other risk factors of suicidality in eating disorders

The literature described by Franko and Keel in their 2006 review as well as more recent publications identified in this review consistently emphasize the clinical importance of identifiable risk factors associated with suicidality in EDs. Individual studies have identified a range of factors that may be associated with increased risk of suicidality and/or associated behaviors among individuals with EDs. Illness severity [26], co-occurring psychiatric illnesses including borderline personality disorder [17] and substance abuse [25], excessive exercise [60], and alexithymia [16] have been mentioned as possible risk factors. With regard to age groups, Dancyger and Fomari found that across ED diagnoses, adolescents demonstrate a strong relationship between suicidal behavior and completed suicides [21]. Dancyger and Fomari’s review concluded with an emphasis on the importance of clinical awareness to the heightened risk of suicide in the adolescent ED population, the treatment for which may present to be challenging as it requires careful coordination between clinicians, parents, and the young adult patients themselves.

#### Self-injurious behaviors

In addition, data from reviews, clinical longitudinal investigations, and community cohort studies, show significant associations between EDs and self-injurious behaviors [18,23,60,64,65]. Self-injurious behaviors, which may include compulsive behaviors (e.g., hair pulling, nail biting, skin picking, self-biting) and impulsive self-injurious behaviors (e.g., cutting, burning, self-hitting, banging, scratching), are sometimes referred to as NSSI because the individuals engaging in the behaviors are doing so with the intention of physical self-harm, but without suicidal intent [23]. However, these behaviors, considered on a spectrum of self-destructive behaviors, may be associated with risk of suicidality [23,65].

Data on the relationship between self-injury and EDs indicate that self-injury has a higher prevalence in ED patients with a history of BN, binge eating, or purging behaviors. Tozzi et al. focused specifically on laxative abuse and found in samples of AN and BN that the function of laxative abuse included both a method of purging as well as a form of self-harm [65]. In a case study of 70 female inpatients with EDs, Claes et al. found that 38.6% engaged in NSSI [18]. Of the NSSI and ED group, a significantly higher portion of individuals with BN engaged in NSSI than did individuals with AN (p < 0.05) [18]. Ahrén-Moonga and colleagues found that 36.8% of a group of 38 ED patients engaged in self-injury [8]. Fourteen of the patients also reported suicidal ideation or plans, and 10 patients had a history of suicide attempts. Consistent with other studies [18,23,60,64,65], these data show a higher prevalence of self-injury—as well as suicide attempts—in BN compared with AN (p < 0.05 and 0.02, respectively) [8].

Other factors that appear to positively influence the relationship between EDs and self-injury include depression, impulsivity, obsessive-compulsiveness, affect dysregulation, dissociation, self-criticizing cognitive style, and need for control, as well as affective lability and previous suicidal behavior or attempts [9,53,64]. Consistent with previous reports, the current review found support for a strong association between EDs and self-injury among individuals with EDs who report a history of trauma and abuse, including verbal, emotional, neglectful, physical, sexual, and/or substance abuse [8,18,24,43,47]. Furthermore, one study that looked specifically at the relationship between pregnancy and self-injury in an ED sample found that, while the majority of women with EDs experience improved ED symptoms during pregnancy, some women in this sample, particularly those diagnosed with binge eating pathology or subclinical ED diagnoses, had a risk of experiencing escalated pathological behaviors during pregnancy [35].

These additional risk factors that may co-occur with or even result in self-injury seem to correlate with more severe ED pathology among ED individuals. In a longitudinal follow-up study, Peterson et al. found that young-adult BN patients who engaged in NSSI at baseline showed an association with increased purging at follow-up eight months after initial presentation [48]. The results also showed that negative urgency at baseline was a significant predictor of NSSI and BN symptoms at follow-up. Fujimori and colleagues found that among individuals with EDs, those who reported self-injury had higher Eating Disorder Inventory scores, signifying greater severity of ED symptoms, than those without self-injury (p < 0.001) [31]. In Muehlenkamp and colleagues’ study of 422 inpatient ED females, 34.6% of participants engaged in NSSI [43]. This investigation revealed that no specific ED diagnosis was associated with self-injury more commonly than another, but the ED types that specifically include binge or purge behaviors (e.g. AN-BP and BN) were associated with a significantly higher number of types of NSSI including cutting, scratching, bruising, and burning, compared to individuals with other ED types (X^2^ (16) = 32.94, P < 0.01) [43]. In an all-male ED sample (N = 130), Claes and colleagues found that 20.8% reported engaging in NSSI [19]. The NSSI group showed significantly more ED symptoms and had an overall more severe presentation than the non-self-injurious ED males. While there was no significant difference in the rate of NSSI occurring among the three ED diagnostic groups in the sample (AN, BN, and EDNOS), there was a tendency for a higher rate of NSSI among the BN group [19].

Self-injurious behaviors are especially common among adolescents with ED symptoms. Even among adolescents without specific ED diagnoses, results indicate that students who engage in NSSI display significantly more eating pathology, including increased body dissatisfaction and bulimic tendencies (especially binge eating), than their non-NSSI peers [6]. Peebles et al. conducted a chart review of 1432 adolescent ED patients over a nine-year period and found that 40.8% of the sample reported engaging in self-injury [47]. Of those ED adolescents reporting self-injury, females were more likely than males to engage in self-harm behaviors, the most common of which was cutting (85.2%). In this sample, there was also a strong association between self-injury and history of abuse (p < 0.05) [47].

## Conclusion

As a group, despite the variation in the content of the existing literature, the 66 publications described in this narrative review support the finding of a consistently high prevalence of suicidality associated with EDs. Specifically, AN is consistently associated with high rates of suicide, especially in the context of significantly elevated SMRs for adolescents and young adults. Contrary to previous findings, some recent studies have found that suicide risk appears elevated in BN as well, although there is more variability in the literature on BN study findings; this may be due to sample differences across studies, including the possibility that some individuals with BN may have a history of AN. Self-injurious behaviors are frequent in EDs, with higher rates among EDs that include binge and purge behaviors. Self-injurious behaviors, which may or may not include suicidal intent, are especially high among adolescents with EDs. Furthermore, recent investigations on individuals who engage in rigorous physical exercise are important because they suggest that over-exercise – often considered a self-injurious behavior among ED individuals – may pose additional risk beyond the physical consequence of the activity itself. Over-exercise may change pain sensation and in turn affect individuals’ acquired capability for suicide. Based on these findings, individuals with EDs, including those with BN symptoms, who engage in over-exercise, may be at higher risk for suicide. These data underscore the importance of assessing for suicide risk not just in individuals with AN, where suicide rates have long been recognized as elevated, but also in individuals presenting with BN and other ED symptoms. Ultimately, there are a number of risk factors associated with suicidality, which may occur across ED diagnoses. Clinicians must pay close attention to these factors, including chronicity of illness, comorbid diagnoses, history of abuse, and self-injurious behaviors, as occurrence of these factors may increase the potential risk of suicidality.

### Limitations

There are several limitations with regard to this review’s assessment of the prevalence of suicidality and self-injury in EDs. Methodologically, the review is narrative without a quantitative aggregation of data. While all authors read the reviews that resulted in the literature search, the reviewing of only papers in English limited the quantity of literature search results reviewed. Additionally, the authors’ search for the DSM-5’s newly described diagnosis ARFID produced no results. Perhaps future published data will include the prevalence of suicidality in EDs, including ARFID, corresponding to newly described criteria in the DSM-5. Ultimately, while more standardized investigation is necessary, multiple studies have clearly found a high risk of mortality, including risk of suicide and self-injurious behaviors, among patients with EDs.

## Abbreviations

AN: Anorexia nervosa
ARFID: Avoidant/restrictive food intake disorder
BED: Binge eating disorder
BN: Bulimia nervosa
CI: Confidence interval
ED: Eating disorder
EDNOS: Eating disorder not otherwise specified
NSSI: Non-suicidal self-injury
OR: Odds ratio
SMR: Standardized mortality ratio
